# Immune dysfunction in patients with obstructive jaundice before and after endoscopic retrograde cholangiopancreatography

**DOI:** 10.1042/CS20160326

**Published:** 2016-07-18

**Authors:** Abeed H. Chowdhury, Miguel Camara, Luisa Martinez-Pomares, Abed M. Zaitoun, Oleg Eremin, Guruprasad P. Aithal, Dileep N. Lobo

**Affiliations:** * National Institute for Health Research Nottingham Digestive Diseases Biomedical Research Unit, Nottingham University Hospitals NHS Trust and University of Nottingham, Queen's Medical Centre, Nottingham NG7 2UH, U.K.; † School of Life Sciences, University of Nottingham Medical School, Nottingham NG7 2UH, U.K.

**Keywords:** cytokines, dendritic cells, duodenal morphology, endotoxin tolerance, immune dysfunction, obstructive jaundice

## Abstract

This prospective observational study investigated monocyte cytokine responses to lipopolysaccharide (LPS) in patients with obstructive jaundice (OJ) before and after endoscopic biliary drainage. Dendritic cell (DC) subsets and their expression of co-stimulatory molecules were also studied. Forty patients with OJ and ten non-jaundiced patients with normal gastroscopy findings were recruited. Ten healthy volunteers provided control blood samples for immunological assays. Patients with OJ had blood and duodenal mucosa sampled at the time of endoscopic retrograde cholangiopancreatography (ERCP) and further blood sampled during the recovery phase. Monocyte cytokine responses to LPS, DC subsets and co-stimulatory molecule expression were compared with controls. Duodenal morphology and occludin expression were also assessed. Monocytes obtained before ERCP from jaundiced patients demonstrated reduced cytokine responses to endotoxin compared with controls (IL-1β: 2678 compared with 4631 pg/ml, *P*=0.04 and IL-6: 3442 compared with 6157 pg/ml, *P*=0.002). Monocytes from patients with malignancy had poorer responses to endotoxin than from those with benign OJ (IL-1β: 2025 compared with 3332 pg/ml, *P*=0.001). After ERCP, the secretion of inflammatory cytokines by monocytes obtained from jaundiced patients increased (IL-1β: 2150 compared with 2520 pg/ml, *P*=0.03 and IL-6: 2488 compared with 3250 pg/ml, *P*=0.01). Occludin expression (85 compared with 95%, *P*=0.004) and mean duodenal villus height (334 compared with 404 μm, *P*=0.03) were lower in jaundiced patients. Before biliary drainage, patients with OJ had a higher percentage of myeloid dendritic cells (mDCs) and greater mDC expression of CD40 (*P*=0.04) and CD86 (*P*=0.04). Monocytes from patients with OJ had lower proinflammatory cytokine secretion in response to LPS, an effect reversed following biliary drainage.

## CLINICAL PERSPECTIVES

• Patients with OJ have higher rates of perioperative sepsis than non-jaundiced patients but the mechanisms underlying this observation remain unclear. ET, which is characterized by the decreased production of inflammatory cytokines by immune cells following exposure to LPS, is a mechanism that may explain this observed elevated risk.
• Compared with healthy individuals, monocytes obtained from patients with OJ had reduced proinflammatory cytokine secretion in response to exposure to LPS. These cytokine secretory responses were restored following relief of OJ.
• Understanding mechanisms of immune cell dysfunction in OJ may indicate potential clinical pathways to reduce the perioperative risk of septic complications in these patients.

## INTRODUCTION

Patients with obstructive jaundice (OJ) are at a higher risk of perioperative complications than those without jaundice [1–3]. They are susceptible to infection and renal impairment, and despite advances in critical care and antimicrobial therapy, mortality rates remain high [4,5]. Although the underlying mechanisms for this elevated risk are not fully understood, evidence suggests that OJ may allow translocation of bacteria or bacterial products into the portal and, subsequently, systemic circulations which might result in chronic activation of immune cells in response to endotoxin exposure. The evidence supporting this hypothesis has come mainly from animal models as it is difficult to sample portal blood or lymphoid tissue in humans [6,7]. These studies confirm impaired intestinal integrity, and translocation of enteric bacteria and bacterial products to mesenteric lymph nodes in the presence of biliary obstruction [6,8]. Diminished capacity of Kupffer cells to clear bacteria or endotoxin from the portal circulation is observed and these effects may contribute to the development of low-grade systemic inflammation which might impair anti-microbial immunity and lead to sepsis or multiple organ dysfunction [9,10]. Bacterial translocation to mesenteric lymph nodes has been demonstrated following laparotomy and in patients with OJ this is associated with a higher rate of post-operative complications [11].

Endotoxin tolerance (ET) is defined as a transient hyporesponsiveness of the host's immune cells to repeated or prolonged exposure to low doses of lipopolysaccharide (LPS) [12]. Originally observed in animals pretreated with low dose LPS, ET is characterized by decreased production of the proinflammatory cytokines tumour necrosis factor-alpha (TNF-α), interleukin-1 beta (IL-1β) and IL-6, as well as lower mortality rates following repeated challenge with LPS [13,14]. Studies in both healthy volunteers and patients with sepsis have provided evidence for ET in humans [15–17]. Monocytes obtained from patients with sepsis demonstrated a reduced capacity for secretion of TNF-α, IL-1β and IL-6 following *ex vivo* stimulation with LPS [18–22]. Conversely, LPS-stimulated monocytic secretion of IL-10 and transforming growth factor-beta (TGF-β) from septic patients remain unaffected, indicating that these cells are still able to respond to LPS but that this response has been altered with a polarization of the T helper (Th) cell profile [23,24]. Dendritic cells (DCs), macrophages and neutrophils have been shown to mirror the tolerance responses of monocytes [25,26]. DCs as key antigen presenting cells, may contribute further to tolerance by driving Th1 responses to a Th2 pattern [27].

It is recognized that inflammatory cascades left unchecked can cause widespread organ dysfunction and ultimately death. ET may be a mechanism to curb this response and induce an anti-inflammatory phenotype with repeated exposure to endotoxin. Hence, ET may contribute to the increased susceptibility to post-operative septic complications seen in patients with OJ.

The aims of this study were to:

- seek evidence of ET in patients with OJ by investigating the capacity of monocytes to generate inflammatory cytokines and the effect of relieving the jaundice by ERCP.
- examine the changes in DC subsets expression of co-stimulatory molecules and alterations in intestinal mucosal architecture in the presence of OJ due to benign and malignant disease.
- study mechanisms rather than outcomes of these phenomena.

## MATERIALS AND METHODS

### Study design and setting

This was a prospective observational study in 40 patients with OJ before and after endoscopic biliary drainage to examine *ex vivo* monocyte responses while jaundiced and during the recovery phase. These were compared with monocyte responses from blood samples obtained from 10 healthy volunteers. Participants were recruited at Nottingham University Hospitals NHS Trust.

### Ethics and consent

The study was granted approval by the UK National Research Ethics Service and the protocol was registered at http://www.clinicaltrials.gov (NCT01367821). Informed written consent was obtained from all participants.

### Selection criteria for study groups

There were four study groups.

#### Groups 1 and 2

Groups 1 and 2 comprised patients with OJ scheduled to undergo ERCP, with 20 patients having malignant biliary obstruction (Group 1) and 20 benign biliary obstruction (Group 2).

*Inclusion criteria:* male or female patients, aged 18–75 years, with evidence of OJ and a serum bilirubin >30 μmol/l.

*Exclusion criteria:* neutropenia (<1.5×10^9^/l), smokers/substance abusers, patients with diabetes, taking corticosteroids, on antibiotics, with active biliary sepsis, who had undergone organ transplantation, with pancreatic pseudocysts, patients with haematological malignancies and any patient who had incomplete biliary drainage or failure of ERCP. Patients were withdrawn from the study if they became critically ill (defined as APACHE II Score ≥8) at any time.

#### Group 3

Ten non-jaundiced patients undergoing oesophagogastroduodenoscopy (OGD) for the investigation of upper gastrointestinal symptoms were recruited to only provide control duodenal mucosal specimens. Patients were excluded if any abnormalities were found at OGD.

#### Group 4

Ten healthy adult volunteers were recruited to provide control blood samples for monocyte assays. Volunteers with any acute illness in the preceding 6 weeks, on any regular medication and those with a history of smoking/substance abuse were excluded.

### Interventions

All patients with OJ underwent treatment of the obstruction at ERCP by stone extraction or stent placement. Non-jaundiced patients underwent OGD. End points studied before and after drainage of bile included serum bilirubin, alanine aminotransferase (ALT), alkaline phosphatase (ALP), white cell count (WCC), C-reactive protein (CRP) concentration, blood monocyte cytokine responses following stimulation with LPS, changes in DC subsets and myeloid (m) mDC co-stimulatory molecule expression. One-off endpoints included culture of bile, duodenal villus height and occludin expression.

### Sample collection and immunological assays

An 80 ml blood sample was obtained from patients with OJ prior to ERCP and used for immunological assays and the measurement of haematological and biochemical parameters. Additional 80 ml blood samples were collected on days 1, 7, 14 and 30 following biliary drainage. If patients were to undergo a further intervention or surgery prior to the 30-day blood sample, the final sampling time point was brought forward. A similar 80 ml blood sample was drawn once from each healthy volunteer but not from non-jaundiced patients undergoing OGD.

Mononuclear cells were isolated from whole blood and monocytes purified using immunomagnetic separation. Monocytes were isolated at intervals after drainage to assess the time course of responses. During a stimulation period of 24 h at 37°C, monocytes were exposed to differing concentrations of LPS (1, 10 and 100 ng/ml) and cytokine secretion (IL-β, IL-6, IL-10 and IL-1RA) was assessed by flow cytometry (Beckmann Coulter, FC500 MPL, Fullerton). Purified monocytes were then stimulated with different concentrations of LPS and incubated at 37°C for 24 h (*t*_stim_).

To characterize DC subsets, PBMCs were isolated from patients at each sampling time point. Details for these techniques are described in the Supplementary Methods section.

### Bile and duodenal sampling

At the time of ERCP, the common bile duct (CBD) was cannulated (without priming with saline or radiocontrast) and a bile sample was aspirated through a sterile catheter and collected into a sterile universal pot. Two duodenal biopsies were taken during ERCP from patients with OJ and non-jaundiced patients undergoing OGD for investigation of upper GI symptoms. The first sample was collected in a sterile container with formalin and the second in a sterile container with sterile water.

Aliquots of bile (100 μl) were cultured on blood agar (BBL, Microbiology Systems) and MacConkey agar (Thermo Fisher Scientific). All plates were examined after 24–48 h of aerobic incubation at 37°C then daily for 7 days for anaerobic culture. Duodenal biopsies obtained at ERCP from patients with OJ and non-jaundiced controls at OGD were processed to measure mucosal thickness, villus height, crypt depth and occludin expression in a blinded manner (Supplementary Methods).

### Statistical analysis

Statistical analysis was performed using the GraphPad Prism v6.0 software (GraphPad Software) using a repeated measures ANOVA design. Data were subjected to D'Agostino and Pearson omnibus normality tests to determine normality of distribution. As the results were distributed normally, data were analysed with the Student *t* test. Correlation was assessed with Pearson's coefficient of correlation and categorical data were analysed with the Chi-square test. Differences were considered significant at *P*<0.05.

## RESULTS

### Demographics

The demographic profile of the participants is shown in Table 1. Seven patients [five with malignant OJ (three disease-related deaths, two became critically ill); two with benign OJ (became critically ill)] did not complete the study. All patients with malignant OJ had biliary stents placed at ERCP. CBD calculi were extracted in 12 patients with benign OJ, and 8 had biliary stents placed.

**Table 1:** Groups and baseline characteristics of patients in OJ study. Values presented are *n* or mean (S.E.M.) *: *P*<0.05 (benign compared with malignant); ^†^: *P*<0.05 (malignant compared with non-jaundiced).

	Benign obstruction	Malignant obstruction	Non-jaundiced controls	Healthy volunteers
Number of subjects	20	20	10	10
Age (years)	51 (4)	64 (2)*^,^†	47 (1)	36 (1)
Gender (male:female)	9:11	12:8	3:7	6:4
Bilirubin (μmol/l)	151 (16)	196 (24)*	–	–
ALT (IU/l)	63 (7)	112 (11)*	–	–
ALP (IU/l)	106 (14)	164 (17)*	–	–
WCC (×10^9^/l)	12.8 (1.3)	10.5 (0.9)	–	–
CRP (mg/l)	50 (9)	62 (10)*	–	–
Diagnoses/reasons for endoscopy	Choledocholithiasis: 18	Pancreatic cancer: 12	Dyspepsia: 4	
	Benign CBD stricture: 1	Periampullary cancer: 4	Epigastric pain: 4	
	Benign CBD polyp: 1	Malignant CBD stricture: 4	Dysphagia: 2	

### Changes in serum bilirubin, liver enzymes and inflammatory markers in patients with benign and malignant obstruction after drainage

**Figure 1:**
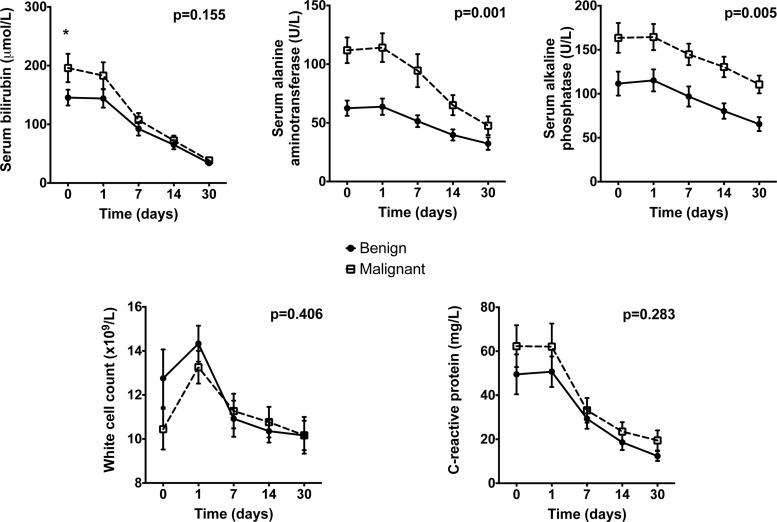
Liver function tests and inflammatory markers Assessment of serum bilirubin, ALT, ALP, WCC and CRP in blood samples from patients undergoing ERCP for OJ. Values presented are mean (S.E.M.). Given *P* values represent test of overall data (repeated measures ANOVA comparing benign with malignant obstruction); *: *P*<0.05 (Student *t* test) for comparison of patients with benign compared with malignant biliary obstruction.

Mean concentrations of serum bilirubin, ALT and ALP were elevated above the reference laboratory ranges in both groups (Figure 1). Although serum bilirubin concentrations were significantly higher in patients with malignant OJ prior to drainage, there was no significant difference between groups after drainage. Both ALT and ALP were higher prior to drainage in patients with malignant OJ compared with those with benign OJ and this difference in concentrations of both enzymes between groups remained after drainage. At the time of recruitment there was a slight, albeit non-significant, elevation in WCC above the normal reference range in patients with benign OJ (Figure 1). In addition, serum CRP concentrations were initially elevated in both groups and reduced significantly after drainage (Figure 1). However, there were no significant differences between patients with benign and malignant OJ in either WCC or CRP.

### Monocyte cytokine responses following stimulation with LPS

**Figure 2:**
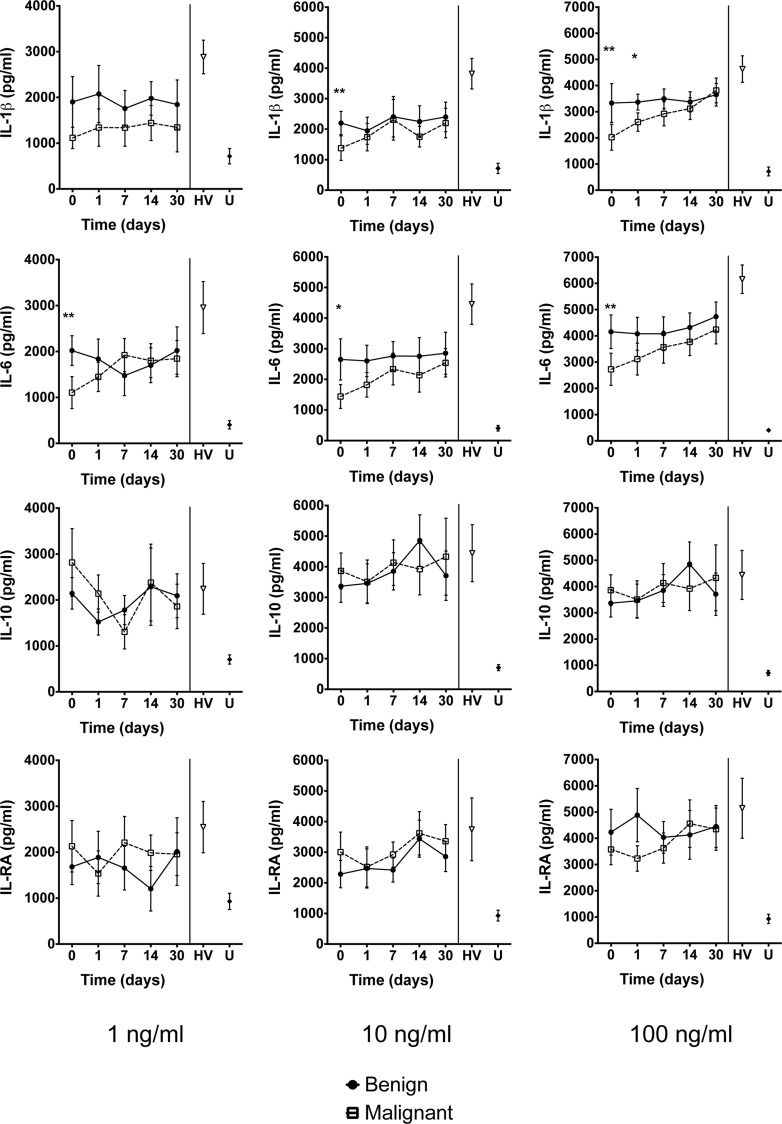
Cytokine responses Flow cytometric assessment of cytokine secretion using the bead array technique following *in vitro* exposure of purified monocytes to 1, 10 and 100 ng/ml LPS for 24 h. Values presented are mean (S.E.M.). HV: healthy volunteers; U: untreated cells. *: *P*<0.05; **: *P*<0.01 (Student *t* test) for comparison of patients with benign compared with malignant biliary obstruction. When incubated with LPS at a concentration of 100 ng/ml, monocytes obtained before ERCP from jaundiced patients demonstrated reduced cytokine responses to endotoxin compared with controls (IL-1β: 2678 compared with 4631 pg/ml, *P*=0.04 and IL-6: 3442 compared with 6157 pg/ml, *P*=0.002). Monocytes from patients with malignancy had poorer responses to endotoxin than from those with benign OJ (IL-1β: 2025 compared with 3332 pg/ml, *P*=0.001). After ERCP, the secretion of inflammatory cytokines by monocytes obtained from jaundiced patients increased (IL-1β: 2150 compared with 2520 pg/ml, *P*=0.03 and IL-6: 2488 compared with 3250 pg/ml, *P*=0.01).

Overall cytokine responses to stimulation with LPS were reduced significantly for IL-1β and IL-6 in patients with OJ compared with healthy volunteers (Figure 2). Prior to biliary drainage, production of IL-6 by monocytes was reduced significantly in patients with malignant OJ compared with monocytes from patients with benign OJ (*P*=0.002). Similar results were observed for stimulation at a concentration of 10 and 100 ng/ml LPS. Monocyte production of IL-10 and IL-1RA was unaffected either before or after biliary drainage and there were no differences between patients with benign and malignant OJ for these cytokines. The capacity for IL-1β and IL-6 production recovered in both the benign and malignant patient groups during the post-drainage period. There was an inverse relationship between serum bilirubin concentrations and monocyte cytokine secretion of IL-1β and IL-6, but this correlation was not statistically significant following Pearson's test.

### Changes in DC numbers following endoscopic biliary drainage

**Figure 3:**
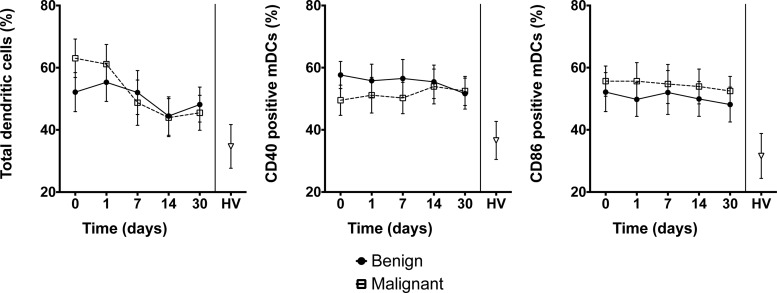
Dendritic cells and co-stimulatory molecules Number of DCs (**left**) and expression of co-stimulatory molecules CD40 (**centre**) and CD86 (**right**) in blood samples from patients with OJ. DCs were selected by flow cytometry using a gate based on HLA-DR^+^Lin1^−^. The mDC population was then selected from a gate demonstrating CD11c^+^CD1c^+^. The co-stimulatory molecules CD40 and CD86 were both selected using the gates SSC^+^CD40^+^ and SSC^+^CD86^+^ respectively. Values presented are mean (S.E.M.). HV: healthy volunteers. *P*>0.05 (repeated measures ANOVA) for comparison of patients with benign compared with malignant biliary obstruction. *P*<0.05 for comparison of healthy volunteers compared with patients with OJ.

Prior to biliary drainage, patients with both benign and malignant biliary obstruction had a higher percentage of myeloid dendritic cells (mDCs) compared with blood samples obtained from 10 healthy controls (*P*<0.05) (Figure 3). However, there was no significant difference between patients with benign and malignant OJ. Following drainage there was a fall in the number of mDCs in patient samples. Compared with healthy volunteers, there was a greater percentage of circulating DCs expressing the activation markers CD40 (*P*=0.04) and CD86 (*P*=0.04) for both patient groups and this higher expression was not affected by biliary drainage (Figure 3).

### Analysis of bacterial organisms in bile

Bile cultures taken at the time of ERCP were analysed for bacterial growth. There was no significant difference in the rate of positive bile cultures (Table 2) between the benign and malignant OJ groups (*P*=0.50).

**Table 2:** Summary of bile culture results in benign and malignant OJ patients

	Number of positive bile cultures (%)	
	Benign obstruction	Malignant obstruction
*Escherichia coli*	4 (20)	3 (15)
*Klebsiella* spp.	2 (10)	–
*Enterobacter* spp.	–	1 (5)
*Pseudomonas aeruginosa*	1 (5)	–
*Enterococcus faecalis*	1 (5)	–
*Streptococcus viridians*	–	1 (5)
**Total**	8 (40)	5 (25)

### Occludin expression and villus height

**Figure 4:**
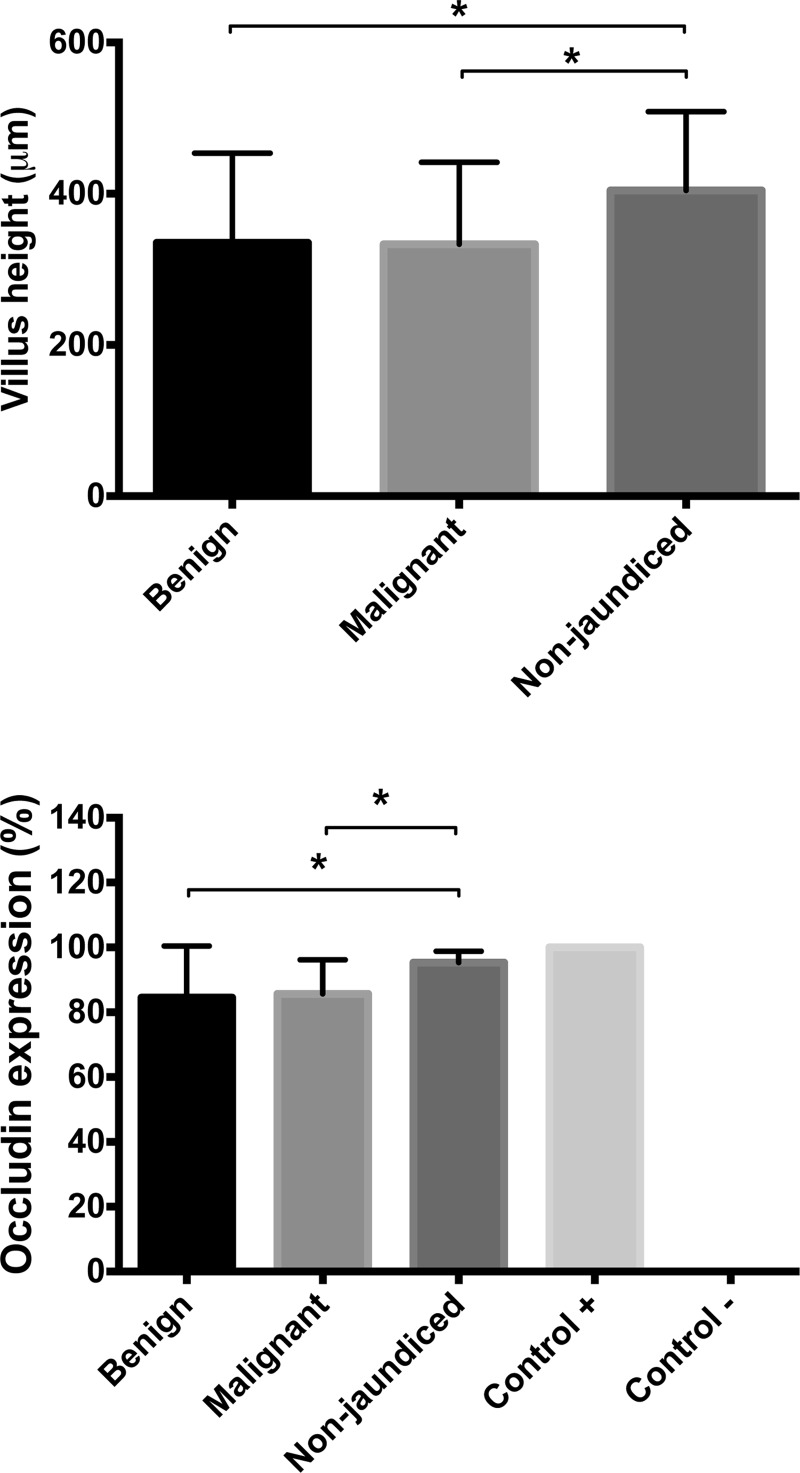
Duodenal morphology Villus height (μm) (**top**) and occludin expression (%) (**bottom**) in duodenal biopsies from patients with benign and malignant OJ and non-jaundiced patients with normal gastroscopy findings. For each patient, 10 randomly chosen mucosal regions were traced using an electromagnetic stylus and villus height measured. For each part of the villus a percentage value of occludin expression was obtained by dividing the number of occludin positive enterocytes by the total number of enterocytes. Values presented are mean (S.E.M.). *: *P*<0.05 (Student *t* test).

Duodenal biopsies taken at the time of ERCP in patients with jaundice demonstrated significant reductions in villus height and occludin expression when compared with controls (Figure 4). However, there were no significant differences in villus height and occludin expression between specimens obtained from patients with benign and malignant jaundice (Figure 4). There was evidence of chronic inflammation in the duodenal biopsies from patients with jaundice which was not seen in specimens obtained from control patients without jaundice.

## DISCUSSION

This study has demonstrated that OJ is associated with diminished monocyte proinflammatory cytokine secretion following exposure to LPS *in vitro*, when compared with healthy controls. In addition, we have shown that monocyte proinflammatory cytokine responses were restored following the relief of biliary obstruction. We have also demonstrated an increased percentage of cells expressing the co-stimulatory molecules CD40 and CD86 on DCs obtained from patients with OJ. A diminished capacity to generate proinflammatory Th1 cytokine responses to bacteria or their products may explain the susceptibility of these patients to perioperative septic complications. Furthermore, demonstration of altered intestinal mucosal architecture in patients with OJ supports the likelihood of bacterial translocation and its potential effects on host immunity and susceptibility to infection.

Compared with monocytes obtained from healthy donors, patients with OJ had a reduced capacity to produce proinflammatory cytokines in response to LPS. This was most evident for LPS concentrations of 100 ng/ml, but for IL-1β and IL-6, lower LPS concentrations produced a similar result. In contrast, *in vitro* monocyte production of IL-10 and IL-RA in response to LPS was unaffected, indicating that suppression was restricted to the generation of proinflammatory cytokines.

One possible mechanism to explain suppression of proinflammatory cytokine production in OJ could involve ET. The clinical implications of this in OJ have not been investigated thus far, but in other conditions such as cystic fibrosis and acute pulmonary syndromes, ET is associated with an increased susceptibility to nosocomial infections [28]. Although ET may protect against septic shock and ischaemia, it is associated with a high incidence of secondary infections. Primary exposure to bacteria or their products during a period of OJ may render immune cells ‘tolerized’ to secondary exposures and limit the magnitude of subsequent cytokine responses, thereby favouring the survival of potential pathogens.

Most studies investigating ET have demonstrated the reduced production of IL-6 and TNF-α [29]. Our results indicate that the proinflammatory cytokine IL-1β may also be affected. Tolerance to endotoxin-stimulated expression of IL-1β has been shown in neutrophils [30] and monocytes [31].

In the present study there was no significant suppressive effect of LPS exposure on monocyte production of the anti-inflammatory cytokine IL-10. Cells obtained from healthy subjects showed a marked increase in secretion of IL-10 compared with untreated cells. Some studies have shown impaired production of IL-10 after previous exposure to LPS [29], whereas others have documented up-regulation of IL-10, TGF-β and IL-1RA [32]. In murine studies, small doses of LPS are lethal in knockout mice unable to transcribe the IL-10 gene [32] and it has been proposed that the IL-10-mediated suppression of TNF-α, IL- 1, IL-6, IL-8 and granulocyte-macrophage colony-stimulating factor by human monocytes may confer a protective effect. Subsequent clinical studies in sepsis have demonstrated the association of ET with high levels of circulating IL-10 and low levels of monocyte human leucocyte antigen - D related (HLA-DR) expression [33].

Patients with malignant biliary obstruction had significantly lower cytokine responses following LPS exposure compared with patients with benign obstruction. In previous studies, higher concentrations of circulating proinflammatory cytokines have been found in patients with malignant obstruction compared with those with benign disease, correlating with elevated acute phase reactants as well as the duration of the jaundice [34,35]. In the few studies that examined circulating cytokine concentrations in patients with OJ undergoing ERCP, low concentrations of IL-6 and TNF-α were observed following drainage [34,36]. The results of the present study indicate that cytokine responses in patients with malignant OJ were impaired to a greater extent when compared with those with benign OJ. The capacity of Kupffer cells to clear LPS may be impaired in the presence of OJ [9,37,38], thus exposing immune cells to higher concentrations of LPS and for prolonged periods. This prolonged exposure to LPS with impaired Kupffer cell function may be one possible mechanism for the differences observed between benign and malignant groups.

Although differences in monocyte cytokine responses were evident between the benign and malignant groups, there were no significant differences in DC phenotype. Compared with healthy volunteers, patients with OJ had a greater proportion of DCs expressing the CD11c cell surface glycoprotein, indicating a predominance of the mDC lineage. Following endoscopic biliary drainage, the proportion of mDCs fell but this was similar in both benign and malignant groups. Previous murine studies have examined the effect of bile duct ligation on liver DC subsets and found a 15-fold expansion of the myeloid lineage [39]. The magnitude of mDC lineage expansion was not of the same order in the present study, although compared with cells obtained from healthy subjects, the proportion of DCs with the CD11c cell surface marker was significantly higher.

On examining co-stimulatory molecule expression on CD11c^+^ cells obtained from patients with OJ, both CD40 and CD86 markers were expressed at higher levels compared with cells obtained from healthy subjects. In murine studies, BDL did not affect the maturation status of DCs [39]. Surface expression of MHC class II, CD40, CD80 and CD86 were similar at 3, 7 and 14 days after BDL, and the ability to induce the proliferation of allogeneic and syngeneic T cells was higher compared with controls [39]. The authors suggested that exposure to LPS may explain this finding as 75% of the mice with bile duct ligation were eventually found to have bacteriobilia.

In the present study, patients with malignant biliary obstruction were significantly more jaundiced than those with benign obstruction prior to intervention. Following ERCP and biliary drainage, however, bilirubin concentrations fell in both groups to reach comparable values. There were no observed differences in degrees of inflammation between malignant and benign groups, as assessed by WCC and CRP. Elevations of WCC and CRP in OJ may be a reaction to bacteraemia occurring after instrumentation or contrast injection, and has been demonstrated previously [40]. Bacteraemia following ERCP is associated rarely with clinical manifestations of sepsis, occurring in only 0.4–0.8% of procedures [41].

In the present study, bacterial growth in bile obtained at ERCP was seen in one third of the patients with OJ, with those with benign obstruction having a slightly higher level of positive cultures, similar to other studies [42,43]. Although it is presumed that organisms ascend to infect static bile, it is also possible that organisms could translocate from the gut into the portal circulation and reach bile through the liver.

Bile exerts trophic effects on the intestinal mucosa, increasing villus density and hypertrophy of intestinal wall components [44]. Recent work has shown a pivotal role of bile in the maintenance of enterocyte tight junctions and the expression of tight junction-associated proteins [44–46]. Weakened integrity of intestinal mucosa could allow passage of bacteria or bacterial products into the portal circulation and our findings could be the consequence of increased exposure to LPS. The results of the present study indicate that OJ induces intestinal morphological changes, such as reduction in villus height and disruption of mucosal architecture. Furthermore, OJ is associated with decreased expression of duodenal occludin compared with non-jaundiced controls. No differences were observed between patients with benign and malignant OJ in either villus height or occludin expression, despite higher bilirubin concentrations in the malignant group. These results are consistent with previous studies in both animals [45,47] and humans [46,48], showing decreased expression of tight-junction proteins in the absence of intestinal bile, which can be reversed once OJ is relieved.

Some limitations of this study warrant discussion. In the study of cytokine responses, the mean age of control subjects was lower than those with disease. It is possible that age-related changes could account for some of the observed differences. However, studies examining the influence of age on proinflammatory cytokine secretion point to an increase in responsiveness to bacterial products with advancing age [49–51]. It might be inferred, therefore, that the differences in cytokine secretion in the present study would likely be wider if control and disease groups were age matched. The changes in cytokine responses have been determined by one modality and it is not clear whether OJ affects receptor function, cytokine expression or secretion. Recently an IL-1R-associated kinase (IRAK-M) has been characterized which acts as a negative regulator in toll like receptor-4 signalling. The significance of this protein is illustrated by the lack of ET in IRAK-M deficient mice [52] and it has been demonstrated that IRAK-M concentrations negatively correlate with the production of TNF-α by mononuclear cells in patients with OJ [53].

In summary, patients with OJ have reduced capacity to generate proinflammatory cytokine responses *in vitro* compared with healthy controls and have changes in intestinal mucosal architecture compared with non-jaundiced individuals. These changes may contribute to the elevated risk of septic complications associated with surgery in these patients.

## Abbreviations

ALP: alkaline phosphatase
ALT: alanine aminotransferase
APACHE: acute physiological assessment and chronic health evaluation
CBD: common bile duct
CD: cluster of differentiation
CRP: C-reactive protein
DC: dendritic cell
ERCP: endoscopic retrograde cholangiopancreatography
ET: endotoxin tolerance
HLA-DR: human leucocyte antigen - D related
IL: interleukin
IRAK: interleukin 1 receptor associated kinase
LPS: lipopolysaccharide
mDCs: myeloid dendritic cells
OGD: oesophagogastroduodenoscopy
OJ: obstructive jaundice
TGF: transforming growth factor
Th: T helper
TNF: tumour necrosis factor
WCC: white cell count
